# The real magic of magic therapy: Improving daily bimanual task performance in children with unilateral spastic cerebral palsy

**DOI:** 10.1111/dmcn.16052

**Published:** 2024-08-12

**Authors:** Richard Wiseman

**Affiliations:** ^1^ Psychology University of Hertfordshire Hatfield UK

## Abstract

This commentary is on the systematic review by Lee et al. on pages 49–58 of this issue.

Learning how to perform magic is fun and interesting. However, there is more to this fascinating activity than meets the eye. The systematic review by Lee et al.^1^ is a welcome addition to the growing literature examining how entertainment magic boosts well‐being and learning.^2^, ^3^, ^4^ The authors examined whether teaching magic tricks to children with unilateral cerebral palsy helps them to carry out daily bimanual tasks. The paper identified four key studies, and the combined results of this work suggested that magic‐based interventions do indeed have a significant and positive impact.

These findings will be of interest to a broad range of health practitioners, including those exploring the link between the performing arts and well‐being. The use of magic in health‐related settings is especially important because it has several practical advantages over more traditional types of the performing arts, such as drama, music, and dance.^4^ For example, whereas plays and dance often need to be staged in formal settings, magic can be performed almost anywhere and to any size of audience. Also, whilst learning a musical instrument or memorizing a play can be time‐consuming and challenging, some magic tricks can be mastered very easily and quickly. Magic is highly economical because it can be performed with inexpensive everyday objects, and it is easy for practitioners to select tricks that match individuals' needs and abilities. Finally, most children are attracted to the idea of becoming a magician and so are highly motivated to be involved in this type of intervention.

Of course, like all research, this work is not without its limitations. The review involved just four studies, and the real‐world nature of this work sometimes resulted in a lack of methodological rigor. On the upside, the review revealed that magic‐based training delivered via pre‐recorded videos and online interaction was as effective as magic‐based training presented in person, and that longer training sessions had greater impact. These findings are especially exciting as they suggest that in the future it might be possible to use the internet to deliver effective magic‐based training at scale.

Work exploring the relationship between the performing arts and well‐being involves practitioners and researchers collaborating with experienced performers. In the studies involved in this review, highly skilled magicians often helped to create and deliver the magic‐based sessions. As a result, it is important to acknowledge that the positive findings will be due, at least in part, to their deep understanding and experience. There are no formal qualifications associated with being a magician, and therefore it is important that future work continues to involve performers who have the knowledge to identify suitable tricks and the experience to teach them in a way that is both fun and engaging.

Hopefully, this review will encourage other researchers, health practitioners, and magicians to collaborate and to carry out additional work in this fascinating area. The conjuring arts can have a positive impact on many aspects of well‐being, and we have yet to tap the full potential of this magical way of promoting both our physical and psychological health.

## Data Availability

Not required.
